# Circulating Angiogenic Factors as Biomarkers of Disease Severity and Bacterial Burden in Pulmonary Tuberculosis

**DOI:** 10.1371/journal.pone.0146318

**Published:** 2016-01-04

**Authors:** Nathella Pavan Kumar, Vaithilingam V. Banurekha, Dina Nair, Subash Babu

**Affiliations:** 1 National Institutes of Health—NIRT—International Center for Excellence in Research, Chennai, India; 2 National Institute for Research in Tuberculosis, Chennai, India; University of Bari Medical School, ITALY

## Abstract

**Background:**

Angiogenesis and lymphangiogenesis are classical features of granuloma formation in pulmonary tuberculosis (PTB). In addition, the angiogenic factor—VEGF-A is a known biomarker for PTB.

**Aims/Methodology:**

To examine the association of circulating angiogenic factors with PTB, we examined the systemic levels of VEGF-A, VEGF-C, VEGF-D, VEGF-R1, VEGF-R2 and VEGF-R3in individuals with PTB, latent TB (LTB) or no TB infection (NTB).

**Results:**

Circulating levels of VEGF-A, VEGF-C andVEGF-R2 were significantly higher in PTB compared to LTB or NTB individuals. Moreover, the levels of VEGF-A, VEGF-C and VEGF-R2 were significantly higher in PTB with bilateral and/or cavitary disease. The levels of these factors also exhibited a significant positive relationship with bacterial burdens in PTB. ROC analysis revealed VEGF-A and VEGF-R2 as markers distinguishing PTB from LTB or NTB. Finally, the circulating levels of all the angiogenic factors examined were significantly reduced following successful chemotherapy.

**Conclusion:**

Therefore, our data demonstrate that PTB is associated with elevated levels of circulating angiogenic factors, possibly reflecting vascular and endothelial dysfunction. In addition, some of these circulating angiogenic factors could prove useful as biomarkers to monitor disease severity, bacterial burden and therapeutic responses.

## Introduction

Granulomatous inflammation is characteristic of many autoimmune and infectious diseases [1,2]. The tuberculous granuloma, a central feature in mycobacterial infection, is the hallmark structure of tuberculosis (TB) infection and disease [1,2]. These granulomas are usually characterized by the concomitant development of hypoxia, which in turn acts as stimulus for vascularization [3]. Vascularization in animal models of TB has been shown to be mediated by angiogenesis and lymphangiogenesis [4]. While the primary role of vascularization of the granulomas could be to establish a pathway for transport of immune cells within the structure, angiogenesis could also confer benefit to the growth of *Mycobacterium tuberculosis* (Mtb) within the granuloma or to its spread to distal sites [3]. Moreover, recent evidence using the rabbit and zebrafish models of mycobacterial infection suggests that blockade of host angiogenic signaling results in improved treatment outcomes as well as diminished mycobacterial growth [5,6]. Finally, lymphangiogenesis stimulated by mycobacterial infection has been shown to promote systemic T cell responses against TB infection [7]. Thus, angiogenesis and lymphangiogenesis appear to be crucial players in the pathogenesis of TB.

Vascular endothelial growth factors and their endothelial tyrosine kinase receptors are central regulators of angiogenesis and lymphangiogenesis [8]. The vascular endothelial growth factor (VEGF) family includes five members: VEGF-A, VEGF-B, VEGF-C, VEGF-D and placenta growth factor (PIGF) [8]. These factors bind with differing specificities to three mostly endothelial transmembrane receptors—VEGF-R1, VEGF-R2 and VEGF-R3 [9]. VEGF-A signaling via VEGF-R2 is the major angiogenic pathway, while VEGF-R1 appears to act as a negative regulator of VEGF—mediated angiogenesis [9]. VEGF-B has been shown to be angiogenic in pathological settings [8]. VEGF-C and VEGF-D are the main players in lymphangiogenesis and signal through VEGF-R3 [10]. In addition to lymphangiogenesis, VEGF-R3 also contributes to angiogenesis [10].

VEGF-A has been shown to be elevated in both sputum and peripheral blood of individuals with pulmonary TB (PTB) and has been characterized as an accurate biomarker distinguishing active disease from latent infection [11,12,13,14,15,16]. However, the role of the other systemic angiogenic factors in human TB has never been explored. We, therefore, examined the circulating levels of these angiogenic factors in individuals with PTB, latent TB (LTB) and no TB infection (NTB). Our data reveal a significant association of systemic levels of VEGF-A, VEGF-C and VEGF-R2 with disease severity and bacterial burden and a significant ability of VEGF-A andVEGF-R2 to distinguish PTB from LTB or NTB. Our data also suggest that the factors mentioned above could serve as accurate biomarkers for monitoring therapeutic responses.

## Materials and Methods

### Ethics statement

All individuals were examined as part of a clinical research protocol (NCT01154959) approved by Institutional Review Board of the National Institute for Research in Tuberculosis, and informed written consent was obtained from all participants.

### Study population

Platelet-poor plasma samples from 44 individuals with active pulmonary TB (PTB), 44 individuals with latent TB (LTB) and 44 individuals with no TB (NTB) recruited in Chennai, India. Platelet poor plasma was used since VEGF is known to be released from platelets during platelet aggregation [17]. Platelet poor plasma from sodium-citrated whole blood was collected as previously described [18]. Patients enrolled in the study did not take any drugs interfering with platelet activation or aggregation. The diagnosis of PTB was based on smear and culture positivity. Chest X-rays were used to determine cavitary disease as well as unilateral versus bilateral involvement. Smear grades were used to determine bacterial burdens and classified as 1+, 2+ and 3+. At the time of enrollment, all active TB cases had no record of prior TB disease or ATT. LTB diagnosis was based on TST and Quantiferon TB-Gold ELISA positivity, absence of chest radiograph abnormalities or pulmonary symptoms and negative sputum smears. A positive TST result was defined as an induration at the site of tuberculin inoculation of at least 12 mm in diameter to minimize false positivity due to exposure to environmental mycobacteria. NTB individuals were asymptomatic with normal chest X-rays, negative TST (indurations <5 mm in diameter) and Quantiferon results. All participants were BCG vaccinated, HIV negative, non-diabetic and had normal body mass index. All participants did not exhibit signs or symptoms of any associated lung or systemic disease. The study groups were similar with regard to age and gender and the baseline characteristics of the study participants are shown in Table 1. Standard anti-TB treatment (ATT) was administered to PTB individuals using the directly observed treatment, short course (DOTS) strategy. At 6 months following ATT initiation, fresh plasma samples were obtained. All PTB individuals were culture negative at the end of ATT.

**Table 1:** Demographics of the study groups.

Study Demographics	Pulmonary TB	Latent TB	Non TB
**No. of subjects recruited**	**42**	**44**	**44**
**Gender (M/F)**	**25/17**	**25/19**	**24/20**
**Median Age (Range)**	**35 (18–60)**	**37 (20–60)**	**37 (19–60)**
**Smear Grade (1+ /2+ /3+)**	**13/11/18**	** Negative**	**Not done **
**Quantiferon Gold in Tube**	**Negative**	**Positive**	**Negative**
**Mantoux skin test, mm**	** Not done**	**>12mm**	**<5mm**

### ELISA

Circulating levels of VEGF-A, VEGF-C, VEGF-R1, VEGF-R2 and VEGF-R3 were measured in platelet-poor plasma using the Duoset ELISA Development System (R&D Systems). Quantikine ELISA kit (R&D Systems) was used for measuring VEGF-D in platelet-poor plasma. The lowest detection limits were as follows: VEGF-A, 31.25 pg/mL; VEGF-C, 62.5 pg/mL; VEGF-D, 62.5 pg/mL; VEGF-R1, 125 pg/mL; VEGF-R2, 31.25 pg/mL and VEGF-R3, 156.25 pg/ml.

### Statistical Analysis

Geometric means (GM) were used for measurements of central tendency. Statistically significant differences between the three groups were analyzed using the Kruskal-Wallis test with Dunn’s multiple comparisons. The Mann-Whitney test was used to compare angiogenic factor concentrations between the individuals with pulmonary TB with unilateral or bilateral lung lesions or cavitary or non-cavitary disease. Linear trend post-test was used to compare angiogenic factor concentrations with smear grades (reflecting bacterial burdens). Receiver Operator Characteristics (ROC) curves were designed to test the power of each candidate angiogenic factor to distinguish LTB or NTB from PTB. Wilcoxon signed rank test was used to compare angiogenic factor concentrations before and after ATT. Analyses were performed using GraphPad PRISM Version 5.01.

## Results

### Circulating angiogenic factor levels are elevated in PTB

**Fig 1:**
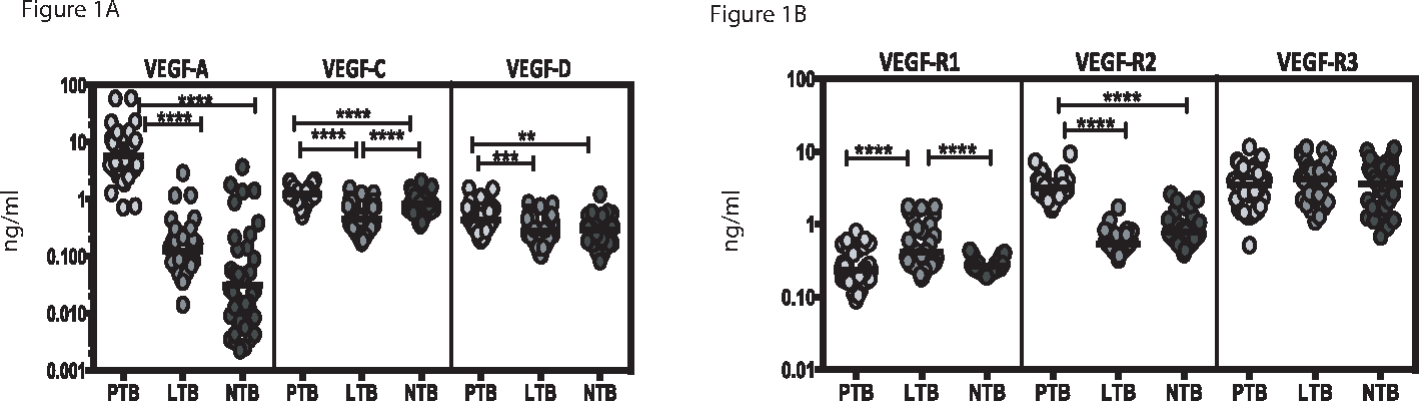
Elevated systemic levels of circulating angiogenic factors in PTB individuals. The plasma levels of (A) vascular endothelial growth factors (VEGF-A, C and D) and (B) VEGF-receptors (VEGF-R1, R2 and R3) were measured in PTB (n = 44), LTB (n = 44) and NTB (n = 44) individuals. The data are represented as scatter plots with each circle representing a single individual. P values were calculated using the Kruskal-Wallis test with Dunn's post hoc comparison.

To determine the systemic levels of angiogenic factors in TB infection and disease, we measured the circulating levels of VEGF-A, C, D, R1, R2 and R3 in PTB, LTB and NTB individuals (Fig 1). As shown in Fig 1A, the systemic levels of VEGF-A (Geometric Mean of 5.85 ng/ml in PTB versus 0.12 ng/ml in LTB and 0.31 ng/ml in NTB), VEGF-C (GM of 1.22 ng/ml in PTB versus 0.46 ng/ml in LTB and 0.83 ng/ml in NTB) and VEGF-D (GM of 0.43 ng/ml in PTB versus 0.27 ng/ml in LTB and 0.29 ng/ml in NTB) were significantly higher in PTB compared to both LTB and NTB individuals. Similarly, the circulating levels of VEGF-R2 (GM of 3.19 ng/ml in PTB versus 0.53 ng/ml in LTB and 0.77 ng/ml in NTB) but not VEGF-R3 was significantly higher in PTB compared to LTB and NTB individuals (Fig 1B). In contrast, the systemic levels of VEGF-R1 (GM of 0.23 ng/ml in PTB versus 0.41 ng/ml in LTB and 0.26 ng/ml in NTB) was significantly higher in LTB compared to both PTB and NTB individuals. Thus, PTB is associated with elevated systemic levels of circulating angiogenic factors.

### Circulating angiogenic factors are markers of disease severity in PTB

**Fig 2:**
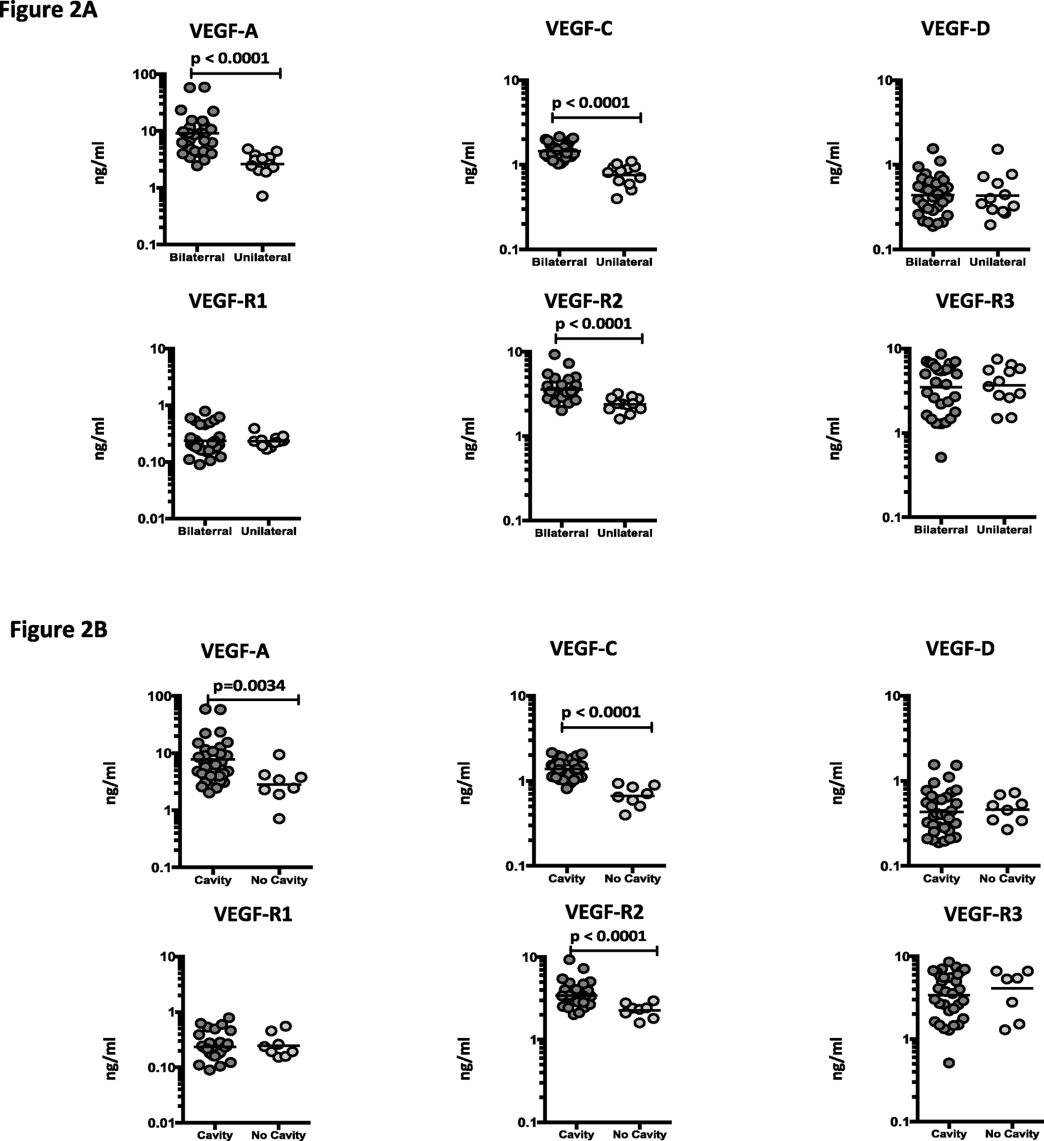
Elevated systemic levels of VEGF-A, VEGF-C and VEGF-R2 in bilateral and cavitary disease in PTB individuals. (A) The plasma levels of VEGF-A, C and D, VEGF-R1, R2 and R3 were measured in PTB individuals with bilateral versus unilateral disease. (B) The plasma levels of VEGF-A, C and D, VEGF-R1, R2 and R3 were measured in PTB individuals with cavitary versus non-cavitary disease. The data are represented as scatter plots with each circle representing a single individual. P values were calculated using the Mann-Whitney test.

To determine the association between the systemic levels of angiogenic factors and disease severity in PTB, we measured the circulating levels of VEGF-A, C, D, R1, R2 and R3 in PTB individuals with unilateral versus bilateral disease and with cavitary versus non-cavitary disease (Fig 2). As shown in Fig 2A, the systemic levels of VEGF-A (GM of 9.06 ng/ml in bilateral vs. 2.63 ng/ml in unilateral disease), VEGF-C (GM of 1.44 ng/ml in bilateral vs. 0.75 ng/ml in unilateral disease) and VEGF-R2 (GM of 3.56 ng/ml in bilateral vs. 2.37 ng/ml in unilateral disease) were significantly higher in PTB individuals with bilateral disease compared to unilateral disease. Similarly, as shown in Fig 2B, the circulating levels of VEGF-A (GM of 7.72 ng/ml in cavitary vs. 2.81 ng/ml in non-cavitary disease), VEGF-C (GM of 1.38 ng/ml in cavitary vs. 0.66 ng/ml in non-cavitary disease) and VEGF-R2 (GM of 7.72 ng/ml in cavitary vs. 2.26 ng/ml in non-cavitary disease) were significantly higher in PTB individuals with cavitary disease compared to those without cavitary disease. Thus, disease severity in PTB is associated with elevated systemic levels of circulating angiogenic factors.

### Circulating angiogenic factors are markers of bacterial burdens in PTB

**Fig 3:**
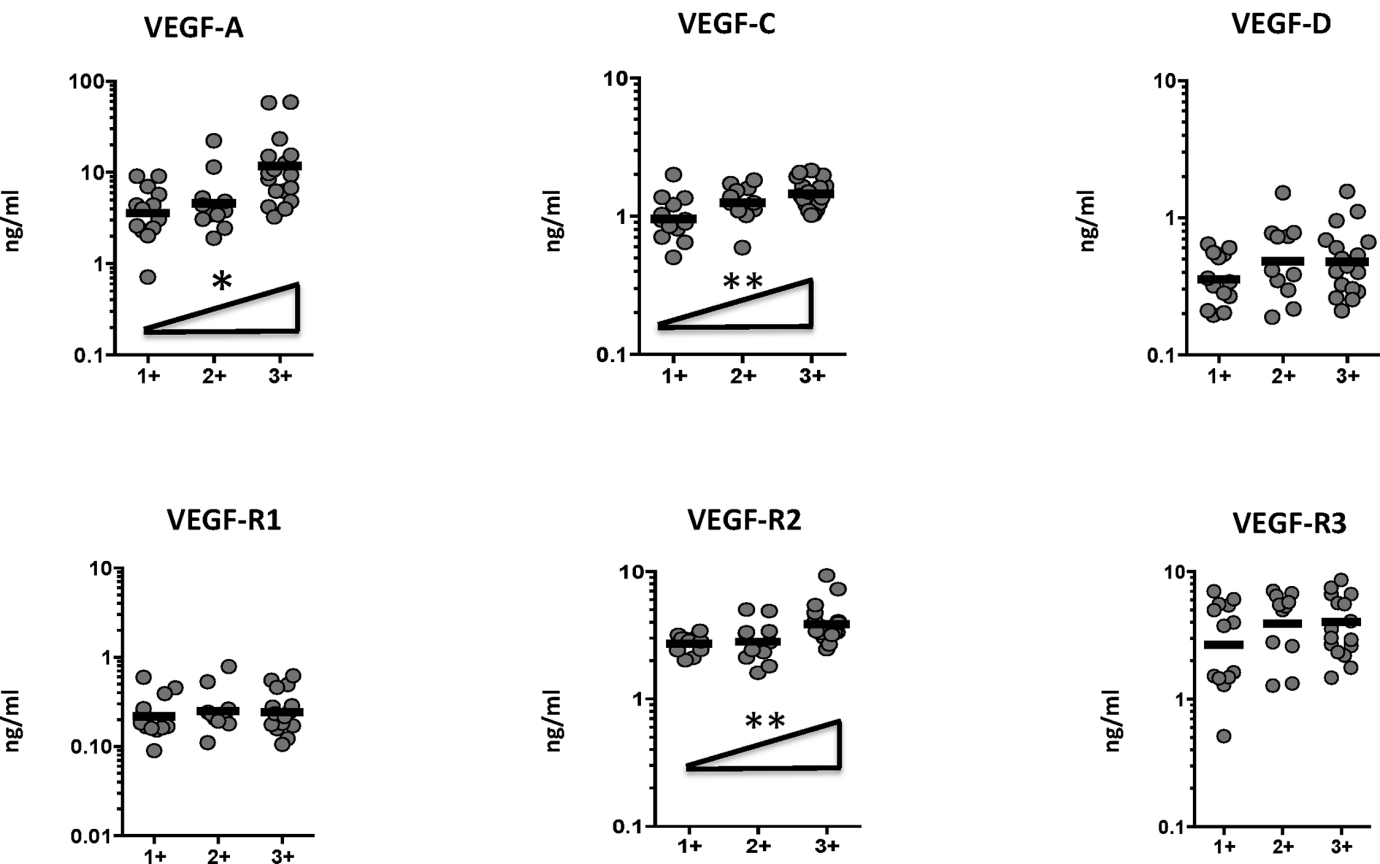
Positive relationship between systemic levels of angiogenic factors and smear grades in PTB individuals. The relationship between the plasma levels of VEGF-A, C, D, VEGF-R1, R2, R3 and smear grades as estimated by sputum smears was examined in all PTB individuals. The data are represented as scatter plots with each circle representing a single individual. P values were calculated using Linear trend post-test.

To determine the association between the systemic levels of angiogenic factors and bacterial burden in PTB, we correlated the circulating levels of VEGF-A, C, D, R1, R2 and R3 in PTB individuals with smear grade classified as 1+, 2+ and 3+ (Fig 3). As shown, the systemic levels of VEGF-A, VEGF-C and VEGF-R2 exhibited a significant positive correlation with smear grades in PTB individuals, indicating a positive association of these factors with bacterial burdens.

### Circulating angiogenic factors can distinguish PTB from LTB and NTB

**Fig 4:**
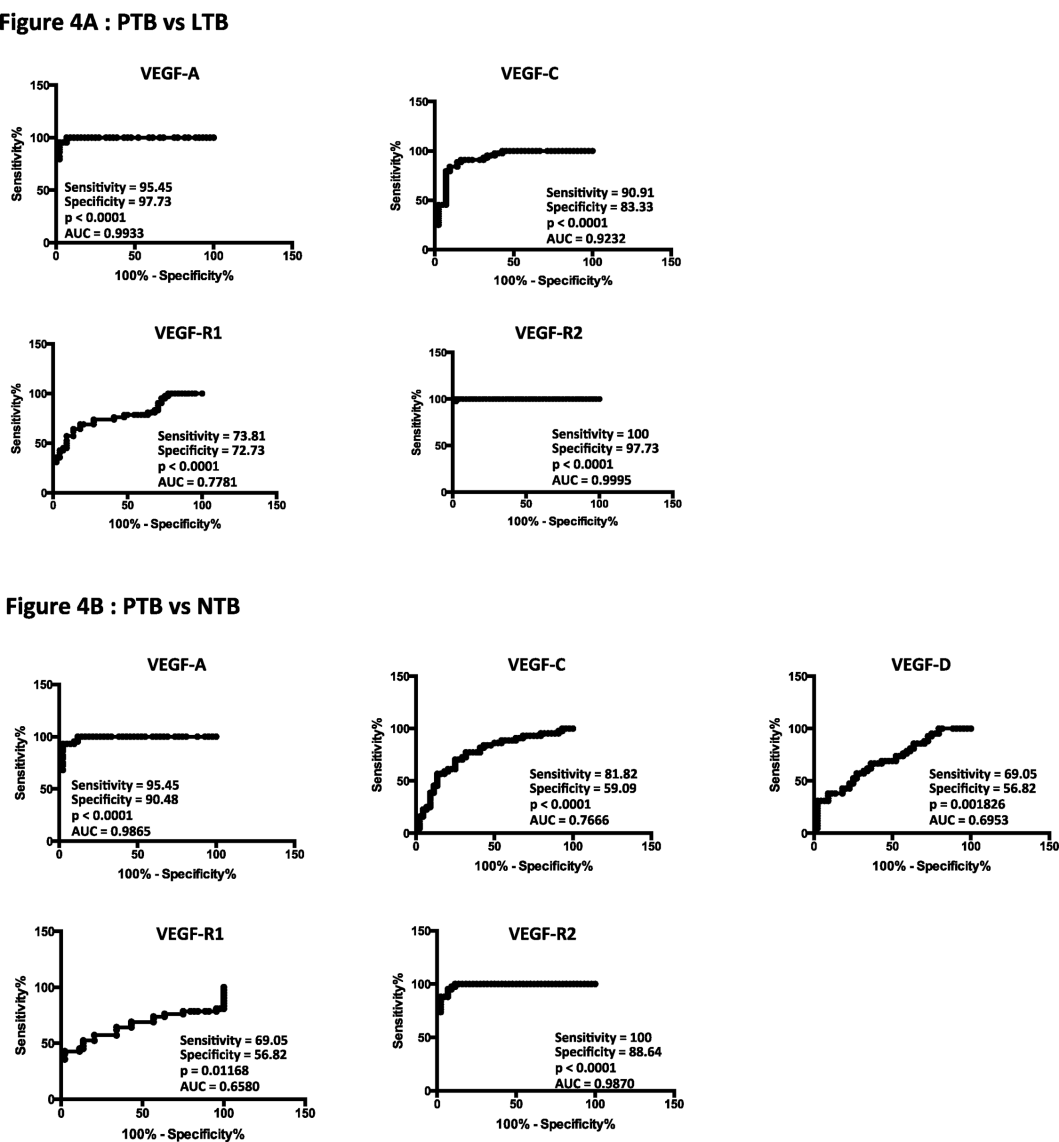
ROC analysis to estimate the discriminatory power of systemic angiogenic factors in PTB individuals. (A) ROC analysis to estimate the sensitivity, specificity and area under the curve was performed using all the systemic angiogenic factors (VEGF-A, C, VEGF-R1, R2 and R3) to estimate the capacity of these factors to distinguish PTB versus LTB individuals. (B) ROC analysis to estimate the sensitivity, specificity and area under the curve was performed using all the systemic angiogenic factors (VEGF-A, C and D, VEGF-R1, R2 and R3) to estimate the capacity of these factors to distinguish PTB versus NTB individuals.

To determine the discriminatory power of circulating angiogenic factor in distinguishing PTB from LTB and NTB, we performed ROC analysis of VEGF-A, C, D, R1, R2 and R3 in PTB versus LTB and PTB versus NTB individuals (Fig 4). As shown in Fig 4A, while most of the angiogenic factors exhibited significant discriminatory power with high area under the curve (AUC) values and sensitivity and specificity, VEGF-A, VEGF-C and VEGF-R2 exhibited the highest AUC and sensitivity and specificity in discriminating PTB from LTB individuals. Similarly as shown in Fig 4B, VEGF-A and VEGF-R2 exhibited the highest AUC and sensitivity and specificity in discriminating PTB from NTB individuals. Thus, VEGF-A and VEGF-R2 exhibit the potential to serve as accurate biomarkers of active TB and to distinguish active TB from infection or no infection.

### Circulating angiogenic factor levels are significantly diminished following ATT

**Fig 5:**
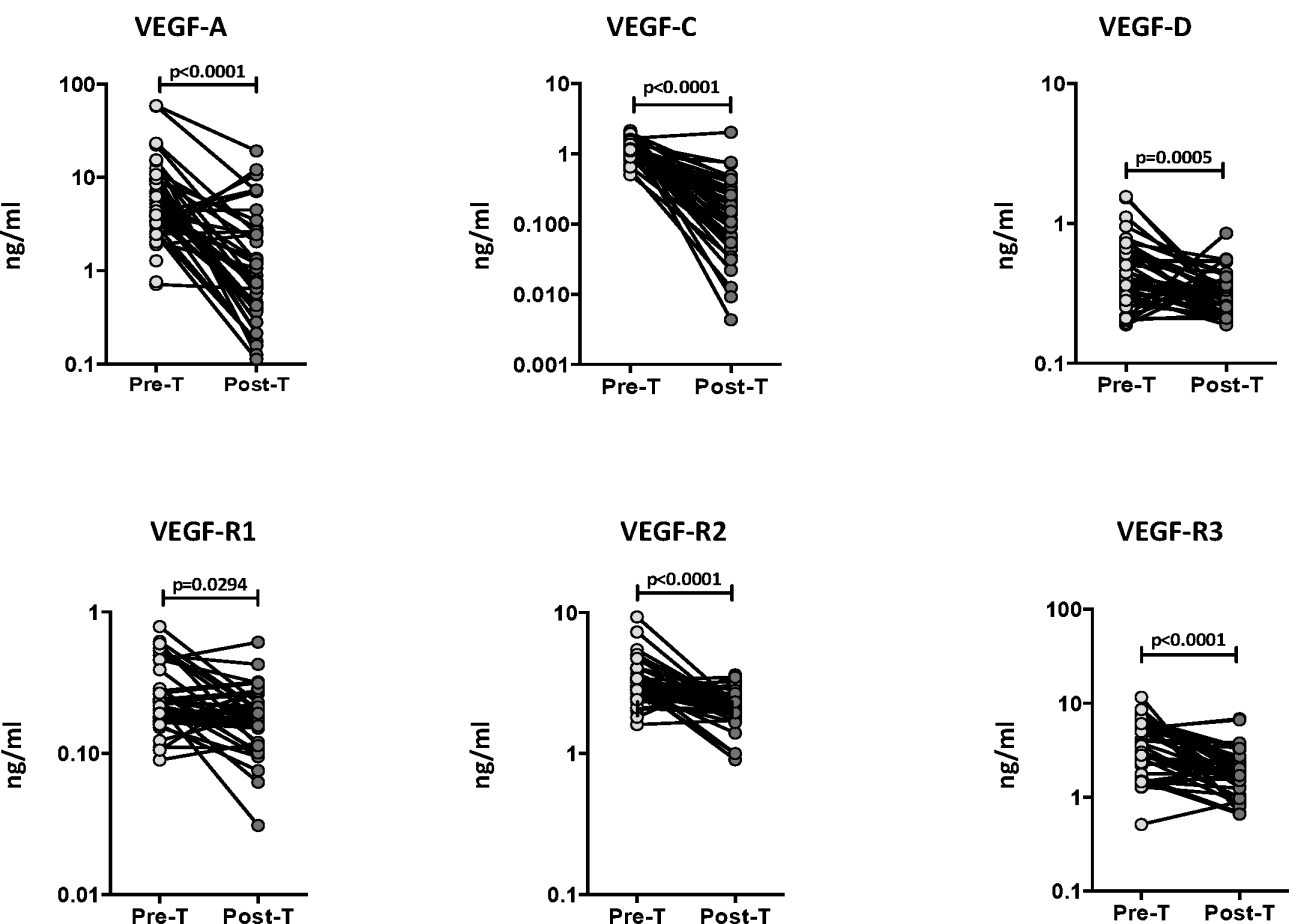
Diminished systemic levels of angiogenic factors following anti-tuberculous treatment in PTB individuals. The plasma levels of VEGF-A, C and D, VEGF-R1, R2 and R3 were measured in PTB individuals before (pre-T) and after (post-T) standard anti-tuberculous chemotherapy. The data are represented as line graphs with each line representing a single individual. P values were calculated using the Wilcoxon signed rank test.

To determine whether the elevated levels of circulating angiogenic factors are directly associated with TB disease, we determined the levels of these factors in PTB individuals before and after a standard course of ATT (pre-T versus post-T). As shown in Fig 5, at the end of ATT, the circulating levels of VEGF-A (GM of 5.85 ng/ml pre-T vs. 1.00 ng/ml post-T), VEGF-C (GM of 1.22 ng/ml pre-T vs. 0.12 ng/ml post-T) and VEGF-D (GM of 0.43 ng/ml pre-T vs. 0.31 ng/ml post-T) were all significantly lower compared to pre-treatment levels. Similarly, the levels of VEGF-R1 (GM of 0.23 ng/ml pre-T vs. 0.17 ng/ml post-T), VEGF-R2 (GM of 3.19 ng/ml pre-T vs. 2.12 ng/ml post-T) and VEGF-R3 (GM of 3.52 ng/ml pre-T vs. 1.83 ng/ml post-T), were also significantly lower post- treatment compared to pre-treatment levels in PTB individuals. Thus, successful treatment of active TB results in significantly diminished levels of circulating angiogenic factors in PTB.

## Discussion

Vascular dysfunction is a major contributor to the pathogenesis of a variety of potentially serious infectious diseases and syndromes, including sepsis and septic shock, hemolytic-uremic syndrome, severe malaria and dengue hemorrhagic fever [19,20]. Because angiogenesis and endothelial activation often precede overt vascular dysfunction, biomarkers of angiogenesis and endothelial activation in plasma may be clinically useful as biomarkers of disease severity or prognosis in infectious diseases [20]. Angiogenesis, lymphangiogenesis and vascular dysfunction are increasingly being recognized as major hallmarks of active TB and utilization of host-directed therapies aimed at blockade of these functions has been proposed as an adjunct measure for TB treatment [5,6]. In addition, the chronic inflammation engendered in active TB disease has been posited to influence angiogenic and lymphangiogenic activity in different models of TB infection [5,6,7]. However, with the exception of VEGF-A and to a lesser extent VEGF-C, the role of circulating angiogenic factors has never been explored in detail in human TB disease.

VEGF-A is a known major player in angiogenesis and lymphangiogenesis and is induced in response to tissue inflammation, hypoxia and pro-inflammatory cytokines [21,22]. Hence, VEGF-A might play a role in blood and lymphatic vessel function by regulating cell trafficking, cellular recruitment and inflammation [21,23]. Indeed, serum VEGF-A levels have been shown to be associated with systemic inflammation in inflammatory lung disease [24] and it has been postulated that VEGF-A might be a major component of the neovascularization of TB granulomas [25]. Moreover, recent evidence from both zebrafish and rabbit models of mycobacterial infection clearly demonstrate an important role for VEGF-A in the pathogenesis of mycobacterial infection [5,6]. Finally, elevated circulating levels of VEGF-A have been reported in pulmonary TB [11,12,13,14,15,16] and VEGF-A appears to be a consistent biomarker of active disease [26]. Our data clearly expand on these reports and confirm that VEGF-A is both a biomarker distinguishing PTB from LTB and NTB with a high degree of sensitivity and specificity as well as a biomarker for therapeutic monitoring, since VEGF-A levels revert to baseline following therapy. In addition, VEGF-A is also an important biomarker of disease severity, extent of disease and bacterial burdens in PTB individuals.

Among the VEGF receptors, only two (VEGF-R2 and VEGF-R3) drive angiogenesis, while VEGF-R1 mostly acts to restrict the angiogenic response [27]. Under homeostasis, stimulation of VEGF-R2 results in angiogenesis of blood vascular endothelial cells, while stimulation of VEGF-R3 elicits a similar response in lymphatic endothelial cells, resulting in lymphangiogenesis [8,9]. Although the VEGF receptors are predominantly expressed on endothelial cells, soluble forms of VEGF receptors are known to be induced in inflammation [9]. Thus, elevated concentrations of VEGF-R2 and R3 reflect underlying angiogenesis or lymphangiogenesis, while elevated levels of VEGF-R1 reflect suppressed angiogenesis [9]. Our study is the first to examine the circulating levels of VEGF receptors in TB and we report that VEGF-R2 is an important and accurate biomarker for differentiating PTB from LTB or NTB and also that elevated circulating levels reflect disease severity and bacterial burdens. In contrast, VEGF-R1 appears to be elevated only in LTB, although its biological significance is yet to be determined. Finally, all the VEGF receptors show decreased plasma levels following treatment indicating that TB disease is intricately associated with angiogenic and lymphangiogenic events in its pathogenesis.

Lymphangiogenesis is known to occur in many human inflammatory diseases, including psoriasis and rheumatoid arthritis, and during transplant rejection [28,29]. Inflammation—induced lymphangiogenesis is thought to regulate fluid drainage, immune cell migration, and removal of inflammatory mediators resulting in resolution of inflammation [29,30]. Conversely, enhanced lymphangiogenesis can induce reactivation of the immune system in the draining lymph node and lead to transplant rejection [29]. A similar scenario has been recently reported in a mouse model of Mtb and *M*. *bovis* BCG, where in, lymphangiogenesis during infection was shown to promote the generation of systemic T cell responses to TB antigens [7]. VEGF-C and VEGF-D are known to modulate lymphangiogenesis via VEGF-R3 signaling [30]. We therefore, examined the circulating levels of the lymphangiogenic factors—VEGF-C, VEGF-D and VEGF-R3. Our data clearly illustrate an important association of active PTB with enhanced levels of VEGF-C, which also serves as an important marker reflecting the extent of pathology and bacterial burden. In addition, the circulating levels of all the lymphangiogenic factors are significantly decreased following treatment.

Our study is the first to systemically explore the association of circulating angiogenic and lymphangiogenic factors in human pulmonary TB and controls. While our study is purely descriptive in nature and does not prove any cause and effect, it nevertheless provides important evidence of association and therefore, the need to explore the role of these factors in pathogenesis further. In addition, since angiogenesis has also been implicated in dissemination of mycobacterial infection, examination of these factors in human extra-pulmonary TB should provide additional input on the pathogenesis of disseminated TB. Moreover, our data lend additional credence to the recent data suggesting that the use of anti-angiogenic agents could have dual benefits in TB disease by both acting as host-directed therapy to directly limit bacterial burden and pathology and also as adjunct treatment to standard anti-TB drugs to either shorten the duration of chemotherapy or treat drug-resistant disease. Finally, our data also add to the growing portfolio of host factors that are of potential use as biomarkers of TB diagnosis and prognosis.
